# The Dynorphin/-Opioid Receptor System at the Interface of Hyperalgesia/Hyperkatifeia and Addiction

**DOI:** 10.1007/s40429-025-00618-x

**Published:** 2025-02-04

**Authors:** Renata C. N. Marchette, Leandro F. Vendruscolo, George F. Koob

**Affiliations:** 1 Neurobiology of Addiction Section, Integrative Neuroscience Research Branch, National Institute on Drug Abuse, Intramural Research Program, National Institutes of Health, BRC Room 08A505.19, 251 Bayview Blvd, Baltimore, MD 21224 USA; 2 Stress and Addiction Neuroscience Unit, Integrative Neuroscience Research Branch, Division of Intramural Clinical and Biological Research, National Institute on Drug Abuse, Intramural Research Program, and National Institute On Alcohol Abuse and Alcoholism, National Institutes of Health, Baltimore, MD 21224 USA

**Keywords:** Opioid withdrawal-induced hyperalgesia, Hyperkatifeia, Dynorphin, Kappa opioid receptor

## Abstract

**Purpose of Review:**

Drug addiction is characterized by compulsive drug seeking and use, accompanied by negative emotional states (hyperkatifeia) and heightened pain sensitivity (hyperalgesia) during withdrawal. Both hyperalgesia and hyperkatifeia are integral components of substance use disorders, negatively impacting treatment and recovery. The underlying neurobiological mechanisms of hyperalgesia and hyperkatifeia involve alterations of brain reward and stress circuits, including the dynorphin/κ-opioid receptor (KOR) system. The dynorphin/KOR system modulates pain perception, negative affect, and addictive behaviors. Here, we review the preclinical evidence of dynorphin/KOR signaling in opioid withdrawal-induced hyperalgesia and hyperkatifeia.

**Recent Findings:**

In opioid dependence models, pharmacological and genetic interventions of the dynorphin/KOR system attenuate somatic and motivational signs of withdrawal and addictive-like behaviors, highlighting its therapeutic potential. Understanding the intricate interplay between dynorphin/KOR signaling, hyperalgesia, hyperkatifeia, and addiction offers novel insights into treatment strategies for opioid use disorder and other substance use disorders.

**Summary:**

Further research is needed to elucidate precise mechanisms of the sexual dimorphism of dynorphin/KOR signaling and identify targeted interventions to mitigate hyperalgesia and hyperkatifeia and facilitate recovery from addiction.

## Introduction

Drug and alcohol addiction are major contributors to disability-adjusted life years throughout the world. For example, the prevalence of alcohol use disorder was the highest of all substance use disorders (1320.8 per 100,000 people), followed by opioid (353.0 per 100,000 people) and cannabis (289.7 per 100,000 people) use disorders [1]. Opioid use disorder, in particular, afflicts over 30 million individuals (or 0.6% of the world’s population) annually, with a similar prevalence in men and women (World Drug Report 2023; United Nations Office on Drugs and Crime: https://dataunodc.un.org/; accessed May 16, 2024). Opioid use disorder can be defined as a compulsion to seek and take opioids, the loss of control over intake, and the emergence of a negative affect state during withdrawal [2, 3]. It can also be considered within an heuristic framework that consists of three stages, with three domains of dysfunction and three neurocircuitries [4–9].

Hyperalgesia and hyperkatifeia are well-documented symptoms of both acute and prolonged opioid withdrawal. These symptoms reflect opponent processes that hold motivational significance [3]. Opioid withdrawal-induced hyperalgesia and opioid withdrawal-induced hyperkatifeia are clinical phenomena whereby patients who chronically use opioids and individuals with opioid use disorder present higher sensitivity and lower tolerance to painful stimuli and greater emotional pain [2]. The International Association for the Study of Pain defines hyperalgesia as greater pain from a stimulus that normally provokes pain, whereas allodynia refers to pain that is experienced from stimuli that normally do not provoke pain (https://www.iasp-pain.org/resources/terminology/; accessed May 16, 2024). Hyperalgesia and allodynia can co-occur in the context of opioid withdrawal. For simplicity, the present review uses the term “hyperalgesia” to encompass sensitized pain responses. Hyperkatifeia, derived from the Greek *katifeia* for dejection or negative emotional state, encompasses the hyper-negative emotional state that is observed during withdrawal, characterized by dysphoria, anxiety, sleep disturbances, irritability, and emotional pain [10].

Hyperalgesia is a common response to chronic opioid use in humans, with tolerance, and even sign-reversal occurring in the presence of opioids [11–14]. Importantly, opioids also alleviate emotional pain, which is a key aspect of hyperkatifeia and a driving force for the withdrawal/negative affect stage of the addiction cycle. Chronic opioid use leads to reduced effectiveness for chronic emotional pain due to withdrawal and tolerance. Indeed, there is a significant contribution of negative affect to the perception of “physical pain” and a significant contribution of “physical pain” to negative affect (see [15]). Under this conceptual framework, we and others have hypothesized that it is the pain and misery of hyperalgesia and hyperkatifeia that drive up the dose requirement during chronic pain treatment [3, 16].

Hyperalgesia and hyperkatifeia are complex constructs that are modulated by biopsychosocial factors. The focus of this review is the potential of dynorphin as a biological mediator of these constructs as evidenced in preclinical research. We also broadly review the state-of-the-art clinical evidence of hyperalgesia and hyperkatifeia before discussing the preclinical literature.

## Clinical Evidence of Hyperalgesia

Opioids are powerful and effective drugs for relieving pain in humans [17], but opioids are less effective with repeated use, such as for the treatment of chronic pain conditions, particularly neuropathic pain, fibromyalgia, and low-back pain. As a result, the efficacy of long-term treatment with opioids is under significant debate, and the risk of prolonged opioid treatment outweighs the benefits in clinical practice [18–20]. In addition, both physical (somatic) symptoms and hard-to-reverse emotional symptoms are observed with established and continuous opioid therapy [3, 7, 10, 18, 21].

Withdrawal from chronic opioid use produces hyperalgesia, an effect that is most prominent in patients with opioid use disorder than in patients with chronic pain [2]. Hyperalgesia has long been observed in people with a history of opioid addiction [22–24]. Heroin users show greater pain sensitivity while in acute withdrawal and in protracted abstinence [23, 25–27]. Patients in methadone-maintenance therapy have low pain tolerance and report more pain [28–33]. In these individuals, pain is a main trigger of relapse or continued opioid use [28, 34]. Moreover, although buprenorphine maintenance therapy may initially improve hyperalgesia, this improvement is not sustained after prolonged exposure [35]. A recent metanalysis showed that patients with chronic opioid use (methadone, buprenorphine) exhibited lower tolerance to pain, and that tolerance to pain was lower right before a methadone dose than it was immediately after a methadone dose [24].

Individuals with opioid use disorder who underwent a 1-month detoxication program exhibited a shorter latency and lower tolerance in a cold pressor test [36]. Former opioid-dependent patients in abstinence (6–38 months) still exhibited abnormal negative emotion in response to the presentation of noxious stimuli [31], thus underscoring the importance of the aversive aspect of pain over the intensity of stimuli. An interaction between the sensorial component of pain (i.e., nociception) and negative emotional aspect of pain was observed in individuals who were in acute withdrawal (24–72 h) from opioids or in protracted abstinence (30 months), in which negative emotion exacerbated hyperalgesia and caused a further increase in pain sensitivity [27].

Studies showed that non-opioid-dependent individuals who underwent acute exposure to opioids also exhibited hyperalgesia upon the cessation of opioid administration [37–39] or upon the precipitation of withdrawal with an opioid receptor antagonist [40], observations that highlight the rapid recruitment of opponent processes that underlie complex analgesia/hyperalgesia responses to opioids.

Pain neurotransmission is modulated by a balance between ascending and descending systems. The ascending system integrates sensory inputs from primary afferents (the dorsal root ganglion and spinal cord) to the somatosensory cortex through two major pathways: spinothalamic pathway and spino-reticulo-thalamic pathway. The descending system, when activated, inhibits pain responses [41]. Such a descending system is composed of projections from the periaqueductal gray (PAG) to the rostral ventromedial medulla to the spinal cord [42]. The descending system can be activated or inhibited by several cortical and subcortical structures, such as the extended amygdala, and is modulated at least partially by endogenous opioid peptides [43]. Opioid withdrawal-induced hyperalgesia is likely mediated centrally because a peripherally restricted opioid receptor antagonist did not precipitate hyperalgesia [44, 45]. Consistent with the central modulation hypothesis, both central sensitization and descending pain modulation were affected by opioids in individuals with hyperalgesia [38, 46].

## Clinical Evidence of Hyperkatifeia

Hyperkatifeia is dissociable from somatic signs of withdrawal and other psychiatric disorders and was described as early as 1880 by Rossbach [47]: “When dependence on opioids finally becomes an illness of itself, opposite effects like restlessness, sleep disturbance, hyperesthesia, neuralgia and irritability become manifest.” Historically, the terms “withdrawal” and “dependence” have been defined differently across various contexts. Withdrawal refers to abstinence from or the cessation of chronic drug use, typically marked by signs and symptoms that are opposite to acute effects of the drug [3].

Dependence is defined as the onset of a withdrawal syndrome when drug use is stopped. According to this definition, any drug, even those without addiction potential, can produce dependence. This concept evolved into the definition of physical dependence, an intense physical disturbance upon discontinuation of drug use. Physical withdrawal syndrome typically presents as opposite effects to that caused by acute administrations of the drug. For example, opioid withdrawal causes pupillary dilation, while opioid intoxication leads to pupillary constriction. Psychological dependence was later defined as a “condition in which a drug produces a feeling of satisfaction and a psychic drive that require periodic or continuous administration of the drug to produce pleasure or to avoid discomfort” [48]. However, both somatic and behavioral symptoms are driven by physiological changes in the body and brain. It is argued that symptoms that are associated with protracted hyperkatifeia hold greater motivational significance for opioid seeking than the somatic signs of withdrawal [3].

This thesis is supported by the strong correlation between dysphoria and spontaneous opioid withdrawal [49]. In studies of heroin-dependent patients undergoing detoxification, the opioid receptor antagonist naloxone increased both somatic signs of withdrawal and dysphoria in a dose-dependent manner [49, 50]. These findings were further supported by community sample studies, which indicated that among individuals with untreated opioid use disorder, withdrawal was perceived as the determining factor in maintaining opioid use and delaying treatment [51]. The author proposed a withdrawal catastrophizing scale as an important clinical measure of hyperkatifeia [52].

A conceptual framework was developed to explain the neural systems thought to mediate hyperkatifeia and drive the motivational aspect of the opponent processes involved in excessive opioid use. This framework involves the downregulation of brain reward circuitry within the system and the recruitment of brain stress circuitry between systems [6, 53]. Within-system neuroadaptation refers to the process where the primary cellular response element to the drug within a specific neurochemical circuit adapts to counteract the effects of the drug. In contrast, between-systems neuroadaptation describes changes in which a different circuit (i.e., stress or antireward circuit) is activated by the reward circuit. The persistence of these opposing effects after the drug is removed is evidenced by the negative emotional withdrawal syndrome.

Consistent with this hyperkatifeia framework, imaging evidence points to an overlap in circuitry that underlies the stress response, reward, and major depressive disorder. Acute stress changes global cortical connectivity and increases striatal connectivity with cortical regions that express genes previously linked to imaging abnormalities in major depression. These regions are abundant in μ- and κ-opioid receptors [54].

## Neurocircuitry of Hyperalgesia and Hyperkatifeia (Pre-Clinical)

In animal models, repeated opioid administration produces a long-lasting, gradual, and dose-dependent decrease in nociceptive thresholds [55–61]. As with human studies, hyperalgesia is a consequence of rapid opioid-induced neuroadaptations and can be observed after a single opioid exposure [62–64].

Opioid withdrawal-induced hyperalgesia has been shown to involve a facilitatory mechanism in a descending system, characterized by greater activity of the rostro ventromedial medulla [65, 66]. Recent studies that used immediate early gene expression markers as a proxy of neuronal activation found greater activity in several subcortical and midbrain regions during hyperalgesia in opioid withdrawal. Hyperalgesia that was precipitated by naloxone following a single morphine exposure increased Fos and Zif268 expression in the central nucleus of the amygdala (CeA) capsular and lateral portions, and amygdalo-striatal transition zone, latero-dorsal bed nucleus of the stria terminalis (BNST), and lateral interstitial nucleus of the posterior limb of the anterior commissure in the brain of male rats[67]. Oxycodone-dependent rats exhibited lower pain thresholds and an increase in Fos-positive neurons in the PAG, CeA, locus coeruleus, paraventricular nucleus of the thalamus, agranular insular cortex, BNST, and lateral habenula medial parvocellular region during withdrawal [68]. In heroin-dependent mice, hyperalgesia was associated with higher Fos expression in the lateral hypothalamus, CeA, ventral tegmental area, parabrachial nucleus, dorsal raphe, and locus coeruleus [60].

Although the term hyperkatifeia was coined relatively recently [10], the construct builds from an extensive body of literature on the manifestation of a negative emotional state or negative affect that is associated with drug withdrawal. Current evidence suggests that the extended amygdala is a focal point in neurocircuitry that mediates hyperkatifeia [69]. Here, within- and between-system neuroadaptations of brain reward and stress systems are hypothesized to drive negative emotional states [3]. Although not part of the extended amygdala, the PAG has reciprocal connections with the extended amygdala, and the PAG integrates negative emotions with autonomic, neuroendocrine, and immune responses [70] and participates in the integration of hyperalgesia and hyperkatifeia [71].

Dynorphin and the κ-opioid receptor (KOR) have been implicated in an array of biological processes, including endocrine regulation [72], neuronal myelinization [73], and itch [74], among others. We review evidence of the involvement of dynorphin/KOR system in domains that underlie hyperalgesia and hyperkatifeia.

### Dynorphin/KOR System

Dynorphins play a role in multiple functional pathways in the brain. They are encoded by the prodynorphin (*Pdyn*) gene that is translated into prodynorphin, a 26 kDa protein, which in turn is processed into big-dynorphin by prohormone convertase 1. Big-dynorphin is further processed by prohormone convertase 2 in the presence of carboxypeptidase E to dynorphin A(1–17), dynorphin A(1–8), dynorphin B, α-neoendorphin, and leumorphin (reviewed by [75–78]). Prodynorphin-derived peptides bind preferentially to the KOR [79].

Prodynorphin is found in most brain structures and generally matches the KOR distribution. Prodynorphin is highly concentrated in the nucleus accumbens, whereas its concentration in the thalamus is low. The KOR is highly expressed in cortical, limbic, and brainstem regions of the rodent brain. Particularly high densities are found in the basal anterior forebrain, including the claustrum, endopiriform cortex, olfactory tubercle, striatum (caudate putamen and nucleus accumbens), preoptic area, hypothalamus, and pituitary [80].

The KOR is a G_i_-coupled receptor that inhibits cell excitability and neurotransmitter release [81]. The activation of KORs by dynorphin leads to lower dopamine transmission [82]. Electrophysiology evidence shows that KOR activation reduces γ-aminobutyric acid (GABA) and glutamate release in the nucleus accumbens [83, 84], glutamate release from the basolateral amygdala to the BNST [85], and glutamate release from the insular cortex to the substantia nigra [86]. In the CeA, the antagonism or genetic deletion of KORs enhanced GABA release that was caused by corticotropin-releasing factor (CRF), suggesting that CRF activation promotes dynorphin release and subsequent KOR activation, which modulates presynaptic GABA release [87].

### Dynorphin and KOR: Pain

Several alterations of the dynorphin/KOR system have been identified in chronic pain. In the spinal cord, the intrathecal administration of high doses of dynorphin produced allodynia in an *N*-methyl-D-aspartate (NMDA)-dependent manner [88, 89], whereas low doses of dynorphin and KOR agonists produced analgesia [90], an effect that was mediated by adenylyl cyclase 3 [91]. Dynorphin knockout mice showed a faster return to normal nociceptive baseline after a peripheral nerve lesion [92], suggesting a pronociceptive role for dynorphin in chronic pain. κ-Opioid receptor antagonism also enhanced allodynia following sciatic nerve injury. The authors proposed that KOR blockade in the spinal cord shifts dynorphin signaling toward a pronociceptive, NMDA-dependent mechanism [93].

Effects of dynorphin in pain conditions are influenced by sexual hormones. In a model of chronic inflammatory pain that was induced by Complete Freund’s Adjuvant administration, hyperalgesia was higher in proestrus, and dynorphin expression was upregulated in the ipsilateral spinal cord in freely cycling females in diestrus and proestrus. The same study showed that prodynorphin expression was upregulated in the spinal cord in ovariectomized females and downregulated in castrated males compared with intact males [94]. However, in the absence of chronic inflammatory pain, systemic treatment with a KOR agonist produced analgesia in male but not female mice, and the lack of analgesia in females was estrogen-dependent [95]. The intrathecal administration of a KOR agonist induced greater analgesia in males and was estrous phase-dependent in females; in gonadectomized rats, estradiol enhanced analgesia and upregulated KOR expression in females but not males, and testosterone had no effect [90]. Further studies are needed to disentangle the role of estrogen in KOR-induced analgesia in acute and chronic pain.

Chronic pain recruits cortical and subcortical structures through ascending and descending pathways. In chronic pain models, there is higher KOR binding in the CeA [96–98]. The administration of KOR antagonists systemically or directly into the amygdala [97], anterior cingulate cortex [99], and nucleus accumbens shell [100] reversed the aversive component of chronic pain, measured by a decrease in the consumption of, or motivation for, palatable substances (e.g., sucrose). Chronic pain upregulated KOR and dynorphin expression in the nucleus accumbens and midbrain and enhanced KOR agonist-induced aversion in male but not female mice. κ-Opioid receptor antagonism prevented pain-induced aversion in males only but reversed chronic pain-induced anxiety- and depressive-like behaviors in both sexes [101]. In female rats, chronic inflammatory pain led to anhedonia- and anxiety-like behavior, and KOR antagonism in the nucleus accumbens prevented the emergence of anxiety-like responses [102]. However, systemic administration of the KOR antagonist JDtic failed to block conditioned place aversion that was produced by visceral and acetic acid-induced pain in mice [103]. In addition to its effects on motivation and negative affect in chronic pain models, KOR antagonism has been shown to reverse sciatic nerve ligation-induced deficits in total, non-rapid-eye-movement, and fragmented sleep [104].

One suggested mechanism for the effects of dynorphin and KORs in the emotional component of pain is the modulation of dopamine transmission. This is evident in chronic pain models, where systemic KOR antagonism blocked the reduction of dopamine release in the nucleus accumbens in inflammatory pain [105]. However, KOR antagonism did not block lactic acid-induced elevations of brain reward thresholds and the decrease in nucleus accumbens dopamine in rats [106].

In summary, despite some contradictory findings, the dynorphin/KOR system is proposed to be an important mediator of negative affect that is associated with chronic pain. The pain modality and dependent variable that is used to measure negative affect are likely important, as well as the dynorphin neuron subcircuits' opposing actions [107, 108].

### Dynorphin and KOR: Negative Affect

Plasma levels of dynorphin positively correlated with the severity of depression [109]. There is an extensive body of work on dysphoric effects of KOR activation and its involvement in depression [110]. The short-acting KOR antagonist aticaprant is being developed for the treatment of anhedonia [111]. The development of KOR antagonists for the treatment of depression is supported by intracranial self-stimulation experiments. κ-Opioid receptor agonists elevated intracranial self-stimulation reward thresholds and decreased the function of the mesolimbic dopamine system [112–116]. κ-Opioid receptor agonists also induced conditioned place aversion [95, 117–119]. κ-Opioid receptor agonism-induced aversion is at least partially mediated by serotonin and dopamine [114, 117, 118] and is less evident in females, an effect that is mediated by estrogen [95]. Anhedonic-like effects of the KOR agonist U50,488 were less evident in females, an effect that was associated with higher tyrosine hydroxylase, an enzyme that is essential for the production of dopamine, in the ventral tegmental area and differential patterns of brain activation, with upregulations in the paraventricular nucleus of the thalamus and BNST [115, 116].

Further evidence of the involvement of KORs in negative affect comes from models of stress-induced anhedonia. κ-Opioid receptor agonism activates the hypothalamic–pituitary–adrenal axis, reflected by an increase in the release of adrenocorticotropic hormone and corticosterone, an increase in the phosphorylation of glucocorticoid receptors, and Fos activation in the thalamus in female rats [120]. Dynorphin and phosphorylated KORs are upregulated in the ventral pallidum and inhibit GABAergic neurons to promote anhedonia-like behavior [121]. The KOR-mediated inhibition of glutamatergic projections from the claustrum to prelimbic cortex was sufficient to promote anhedonia-like behavior [122]. Deleterious effects of chronic social defeat can be prevented by treatment with the long-lasting KOR antagonist norbinaltorphimine [123]. κ-Opioid receptor antagonism also prevented working memory deficits in the face of a stressor in rhesus monkeys [124].

Dynorphin and KORs are also involved in anxiety-like behavior and fear responses. The genetic knockout of KORs selectively in dopaminergic neurons reduced anxiety-like behavior [125]. The deletion of KORs in the CeA and ablation of dynorphin inputs to the CeA increased anxiety-like behavior and impaired conditioned threat discrimination [126]. Dynorphin transmission in the prefrontal cortex suppressed defensive behaviors in response to threat [127]. κ-Opioid receptor stimulation in the caudal portion of the nucleus accumbens shell resulted in the greater inhibition of dopamine release and coincided with the emergence of avoidance behavior [128]. Threat generalization was modulated by KORs in a dorsal raphe-to-ventral tegmental area projection-specific manner [129]. κ-Opioid receptors in the basolateral amygdala regulated anxiety-like behavior and mediated KOR agonist-induced aversion [130]. Upregulation of the dynorphin/KOR system in the amygdala led to the emergence of depression-like behavior following chronic social defeat stress [123]. Altogether, these data suggest the involvement of a dynorphin/KOR system in the dorsal raphe to ventral tegmental area to nucleus accumbens shell and its reciprocal connection with the CeA and basolateral amygdala in the modulation of negative affect.

### Dynorphin and KOR: Drug Addiction

It has been hypothesized that the activation of KORs leads to the negative emotional states that are associated with drug withdrawal [131], pain, and particularly pain associated with acute withdrawal. More specific to opioid dependence, animal studies have demonstrated region-specific increases in dynorphin levels after the passive administration of morphine or heroin in the brain [132, 133] and spinal cord [56, 134, 135]. The precursor of dynorphin, prodynorphin, was upregulated after heroin self-administration [136–138], and dynorphin was also upregulated during the anticipation of heroin self-administration [139].

The genetic deletion of KORs prevented the appearance of naloxone-precipitated withdrawal signs and mitigated social interaction deficits observed during withdrawal in male mice [140]. Administering norbinaltorphimine, a selective antagonist of KOR, 5 h before naltrexone-precipitated withdrawal in morphine-dependent rats decreased certain signs of opioid withdrawal during a 30-min withdrawal session and lowered the subsequent conditioned place aversion to the withdrawal chamber 2 days later [141]. Intraventricular and intra-dorsal hippocampus infusions of norbinaltorphimine blocked naloxone-induced conditioned place aversion when given before naloxone [142]. However, opposite effects were observed when norbinaltorphimine was administered before morphine exposure, and they coincided with a decrease in dopamine release in the nucleus accumbens [143].

The KOR antagonist norbinaltorphimine has been shown to improve myriad opioid withdrawal-related behaviors. Administered systemically or intra-nucleus accumbens shell, norbinaltorphimine reduced the development of addiction-like behavior and anxiety-like behavior during withdrawal from heroin in male rats [138]. In male mice, treatment with norbinaltorphimine was sufficient to prevent and reverse heroin abstinence-induced social avoidance [144] and morphine abstinence-induced anhedonia [145]. Systemic KOR antagonism with either norbinaltorphimine or 5’-guanidinonaltrindole reversed heroin withdrawal-induced hyperalgesia in male and female rats [59], an effect that was recapitulated with an intra-CeA infusion of norbinaltorphimine [146].

The reduction of dopamine release caused by dynorphin, particularly in the nucleus accumbens shell, was also hypothesized to modulate aversive/rewarding aspects of drug intake [147]. Acute and protracted withdrawal from morphine upregulated KORs in the nucleus accumbens. The conditional deletion of KORs in the nucleus accumbens prevented withdrawal-induced anhedonia, and a local norbinaltorphimine infusion reversed it [145]. The KOR agonist U50,488H reduced dopamine release in the nucleus accumbens in rats that self-administered heroin, resulting in an increase in immediate heroin intake [148]. κ-Opioid receptor antagonists did not block the acute rewarding effects of opioids but blocked the stress-induced potentiation of opioid reward, the stress-induced reinstatement of opioid-seeking behavior, and the escalation of opioid intake in an extended access model [138].

Other less extensively studied brain regions that may mediate aversive effects of dynorphin and KORs in opioid dependence include the prefrontal cortex, hippocampus, amygdala, and dorsal raphe. For example, morphine withdrawal caused the release of dynorphin in the prelimbic cortex, leading to working memory deficits [149], whereas the inhibition of a dynorphinergic basolateral amygdala-to-ventral hippocampus projection reversed morphine withdrawal-induced anxiety-like behavior [150]. Morphine abstinence activated dynorphin/KOR signaling in the basolateral amygdala, facilitating glutamate transporter 1 upregulation that led to the inhibition of excitatory inputs to the nucleus accumbens. The activation of basolateral amygdala-to-nucleus accumbens inputs blocked depression-like behavior following morphine abstinence [151].

The dynorphin/KOR modulation of serotonergic transmission has been implicated in negative affect-like effects of opioid withdrawal [144, 152, 153]. Protracted abstinence from morphine led to robust social interaction deficits in mice, and this effect was mediated by KORs in the nucleus accumbens shell and by suppressing the release of serotonin [152].

Dynorphin has long been known to be activated by chronic alcohol administration [154] and chronic psychostimulant administration [6, 155]. For alcohol, polymorphisms of the gene that encodes the KOR (*Opkr1*) have been associated with the severity of alcohol use disorder and depression [156], whereas polymorphisms of the *Pdyn* gene have been associated with alcohol dependence [157] and the propensity to drink in negative emotional states [158], an effect that appears to be more prominent in men [159].

During acute alcohol withdrawal there was an increase in prodynorphin mRNA expression in the CeA [160] and in the nucleus accumbens [161]. Similar findings are seen in the CeA and hypothalamus in alcohol-preferring rats compared with non-preferring rats after voluntary alcohol consumption [162].

Dynorphin signaling is hypothesized to drive behavioral effects of acute withdrawal from alcohol. A KOR antagonist reversed anxiety-like acute withdrawal effects of exposure to chronic, intermittent alcohol exposure in mice [163] and somatic withdrawal and alcohol self-administration in rats [164]. Again, a prominent, proposed mechanism is that alcohol withdrawal exacerbates the inhibition of dopamine release in the nucleus accumbens, which is normalized by KOR antagonism [154, 163, 165, 166].

Dynorphin/KOR signaling in the extended amygdala also has been shown to mediate compulsive-like drinking in alcohol dependence [167]. A combination of a short-acting KOR antagonist and naltrexone decreased voluntary alcohol drinking in mice [168]. Both systemic and intracerebral KOR antagonist administration blocked high compulsive-like alcohol intake [169, 170]. Antagonism of KORs reduced the stress-induced escalation of intake in mice that were exposed to chronic intermittent alcohol [171–173]. The chemogenetic inhibition of KORs in the CeA reduced drinking in alcohol-dependent rats, similar to microinfusions of norbinaltorphimine in the CeA and BNST [174]. The CeA may also mediate effects of KOR blockade on binge-like drinking in mice [173, 175].

The rostro-caudal location of dynorphin and KOR cells in the nucleus accumbens has been shown to bi-directionally modulate alcohol drinking. The stimulation of KORs in the caudal shell stimulated drinking, whereas stimulation in the rostral shell decreased drinking [176]. The bi-directional differences in alcohol intake coincided with the KOR-mediated inhibition of dopamine release in rostral *vs*. caudal portions of the shell [177]. κ-Opioid receptor expression was downregulated in the ventral tegmental area following alcohol exposure [178], and KOR overexpression in ventral tegmental area dopaminergic neurons that project to the nucleus accumbens led to compulsive-like alcohol intake [179]. The nucleus accumbens shell also plays a role in the intersection between pain and alcohol, in which chronic inflammatory pain increased alcohol consumption following abstinence in female rats, an effect that was blocked by KOR antagonism [180].

Other regions have been implicated in actions of dynorphin in addiction-like effects of alcohol. Dynorphin deletion and KOR blockade in the insula reduced the escalation of alcohol intake in a two-bottle choice intermittent-access model [181]. κ-Opioid receptor antagonism in the PAG attenuated alcohol withdrawal-induced anxiety- and depressive-like behaviors [182]. Medial prefrontal cortex KORs mediated working memory deficits in alcohol dependence [183].

Most evidence of the involvement of dynorphin and KORs in the neurobiology of stimulant addiction is related to the regulation of dopamine transmission. Binge-like cocaine self-administration upregulated prodynorphin and KOR expression in the nucleus accumbens and ventral tegmental area and increased KOR agonism-induced dopamine release in the nucleus accumbens [184, 185]. A midbrain dopamine circuit was also shown to modulate cocaine-induced conditioned place preference, dynorphin projections from the dorsal raphe to the ventral tegmental area led to dopamine release in response to cocaine [186]. Systemic [187] and intra-nucleus accumbens core [188] KOR antagonist administration prevented the escalation of cocaine intake in an extended-access model in rats, and these treatments reversed cocaine withdrawal-induced anxiety-like behavior [189]. Extended access to methamphetamine upregulated prodynorphin in the nucleus accumbens core and shell, and systemic and intra-nucleus accumbens shell (but not core) KOR antagonist administration prevented the escalation of methamphetamine intake and decreased the motivation to seek the drug [190]. Methamphetamine withdrawal-induced cognitive deficits may also be mediated by KORs in the hippocampus [191] and prelimbic prefrontal cortex [192].

In nicotine dependence, there was an upregulation of KORs and *Oprk1* expression in the prefrontal cortex, nucleus accumbens, and hippocampus [193]. Moreover, a KOR agonist promoted the reinstatement of nicotine self-administration and conditioned place preference [194–196]. Evidence also indicates that dynorphin and KORs might play a role in cannabis dependence. Withdrawal from chronic systemic exposure to tetrahydrocannabinol upregulated dynorphin and KORs in the nucleus accumbens [197].

## Conclusion

**Fig. 1:**
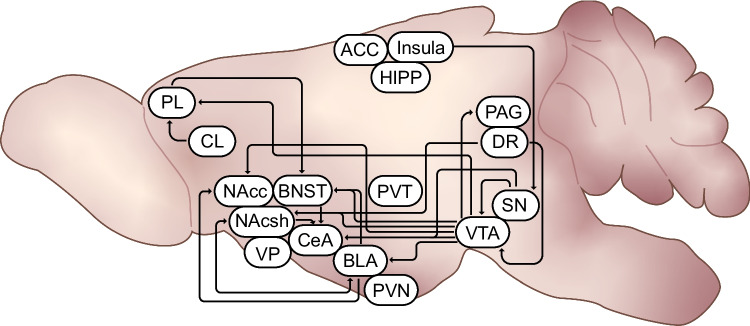
Dynorphin/KOR pathways putatively underlying hyperalgesia and hyperkatifeia. The figure summarizes brain regions and circuits in which dynorphin/KOR signaling has been show to modulate pain, anhedonia, anxiety, motivation, aversion or drug seeking. PL: prelimbic region of prefrontal cortex, CL: claustrum, ACC: anterior cingulate cortex, Insula: insular cortex; HIPP: hippocampus, PAG: periaqueductal grey, DR: dorsal raphe, SN: substantia nigra, VTA: ventral tegmental area, PVN: paraventricular nucleus of the hypothalamus, PVH: paraventricular nucleus of thalamus; CeA: central nucleus of the amygdala, BLA: basolateral amygdala, BNST: bed nucleus of the stria terminalis, NAcc: nucleus accumbens core, NAcsh: nucleus accumbens shell, VP: ventral pallidum

Dynorphin and KORs modulate neurotransmission in the key circuits that are implicated in the interface of chronic pain states, stress, and addiction with a focus on the construct of hyperkatifeia. The circuits and brain regions where KORs and dynorphin modulate behaviors that underlie the hyperalgesia and hyperkatifeia constructs are summarized in Fig. 1. A particular focal point is the modulation of emotional pain or hyperkatifeia by the dynorphin/KOR system via the mesocorticolimbic dopamine system in the context of addiction. Although there is evidence of sexual dimorphism and a role for estrogen in the function of dynorphin and KORs, most published studies have utilized male rodents only. Those that used both male and female subjects frequently presented the data of both sexes combined. In conclusion, we propose that modulation of the dynorphin/KOR system is a promising target for addressing hyperalgesia and hyperkatifeia during opioid withdrawal, which could potentially be used for the treatment of opioid use disorder and addiction more generally.

## Data Availability

No datasets were generated or analyzed during the current study.
